# Opioid reduction and enhanced recovery in orthopaedic surgery (OREOS): A feasibility randomized controlled trial in knee replacement patients

**DOI:** 10.1080/24740527.2022.2102465

**Published:** 2022-10-04

**Authors:** Sushmitha Pallapothu, Kim Madden, Anthony Adili, Adrijana Krsmanovic, Matilda Nowakowski, Tara Packham, Sidra Shoaib, Lehana Thabane, Jean-Eric Tarride, Daniel Tushinski, Harsha Shanthanna

**Affiliations:** a Department of Health Research Methods, McMaster University, Hamilton, Ontario, Canada; b Department of Surgery, McMaster University, Hamilton, Ontario, Canada; c Research Institute of St. Joseph’s Healthcare Hamilton, Hamilton, Ontario, Canada; d Department of Psychiatry and Behavioural Neurosciences, McMaster University, Hamilton, Ontario, Canada; e School of Rehabilitation Science, McMaster University, Hamilton, Ontario, Canada; f Hamilton Health Sciences, Juravinski Hospital, Hamilton, Ontario, Canada; g Department of Anesthesia, McMaster University, Hamilton, Ontario, Canada

## Abstract

**Background:**

Total knee arthroplasties are the second most common surgery in Canada. Most patients recover well, but 20% or more still suffer from persistent pain and opioid use. Though opioids are an important part of perioperative pain management, their potential for long-term adverse effects is well recognized. Limiting opioids may be insufficient to overcome the issue of opioid overuse. Pain and opioid use are highly linked, so an effective alternative needs to address both issues.

**Objectives:**

The principal objective of this pilot trial is to assess the feasibility. The clinical objectives are to determine the effects of a multicomponent care pathway on opioid-free pain control, persisting pain and opioid use, functional knee outcomes, quality of life, and return to function.

**Methods:**

We will include adult patients scheduled for primary elective total knee arthroplasty. Patients in the intervention group will undergo a multicomponent intervention pathway that will be facilitated by an intervention coordinator linking each patient and their surgical/ perioperative team. The interventional pathway will include (1) preoperative education on pain and opioid use, (2) preoperative risk identification and mitigation using cognitive behavioral skills, (3) personalized postdischarge analgesic prescriptions, and (4) continued support for pain control and recovery up to 8 weeks. Patients in the control group will receive the usual care at their institution.

**Discussion:**

The overarching goal is to implement and evaluate a coordinated approach to clinical care to improve pain control and reduce harms, with an emphasis on patient-centered care and shared decision making.

**Trial Registration Number:** NCT04968132 (informed consent/ research ethics board statement).

## RÉSUMÉ

**Contexte:** L'arthroplastie totale du genou est la deuxième chirurgie la plus courante au Canada. La plupart des patients se rétablissent bien, mais au moins 20 pour cent d’entre eux souffrent encore de douleur persistante et de consommation d'opioïdes. Bien que les opioïdes soient un élément important de la prise en charge périopératoire de la douleur, leur potentiel d'effets indésirables à long terme est bien reconnu. La limitation des opioïdes peut être insuffisante pour surmonter le problème de la surconsommation d'opioïdes. La douleur et la consommation d'opioïdes sont étroitement liées, de sorte qu'une option de rechange efficace doit s'attaquer à ces deux problèmes.

**Objectifs:** L'objectif principal de cet essai pilote est d'évaluer la faisabilité. Les objectifs cliniques sont de déterminer les effets d'une voie de soins à composantes multiples sur la maitrise de la douleur sans opioïdes, la douleur persistante et la consommation d'opioïdes, les résultats fonctionnels du genou, la qualité de vie et le retour à la fonction.

**Méthodes**: Nous inclurons les patients adultes devant subir une arthroplastie primaire totale élective du genou. Les patients du groupe d'intervention seront soumis à une voie d'intervention à composantes multiples qui sera facilitée par un coordonnateur d'intervention reliant chaque patient et son équipe chirurgicale/périopératoire. La voie d'intervention comprendra (1) une éducation préopératoire sur la douleur et la consommation d'opioïdes, (2) la détermination et l'atténuation des risques préopératoires à l'aide de compétences comportementales cognitives, (3) des prescriptions analgésiques personnalisées après la sortie, et (4) un soutien continu pour la maîtrise de la douleur et la récupération pendant une période allant jusqu'à huit semaines. Les patients du groupe témoin recevront les soins habituels à leur établissement.

**Discussion**: L'objectif global est de mettre en œuvre et d'évaluer une approche coordonnée des soins cliniques afin d’améliorer la maitrise de la douleur et réduire les méfaits, en mettant l'accent sur les soins centrés sur le patient et la prise de décision partagée. Numéro d'enregistrement de l'essai : NCT04968132 (consentement éclairé/déclaration du comité d'éthique de la recherche).

**Trial Registration Number:** NCT04968132.

